# Supplementary material for: Tracking the popularity and outcomes of all bioRxiv preprints
Source: eLife. 2019 Apr 24;8:e45133. doi: 10.7554/eLife.45133 (PMC6510536; doi:10.7554/eLife.45133)
Supplement: Figure 1—source data 4. [file elife-45133-fig1-data4.docx]

| **Source** | **Articles** |
| --- | --- |
| *Cell* vol. 174(6) | 17 |
| *Cell* vol. 175(1) | 18 |
| *Genetics* vol. 210(1) | 23 |
| *Jour of Biochem* vol. 164(3) | 8 |
| *PLoS Biology* vol. 16(9) | 19 |
| bioRxiv, 1–3 Sep 2018 | 100 |

**Figure 1—source data 4:** The number of full-length articles published by an arbitrary selection of well-known journals in September 2018. The *Cell* count is limited to the "Articles" and "Resources" categories; *Genetics* is limited to their "Investigations" category, and *PLoS Biology* to "Research Articles," "Methods and Resources" and "Meta-Research Articles." Links to each issue’s table of contents are included in supplementary file *figures.md*.
