# Supplementary material for: Tracking the popularity and outcomes of all bioRxiv preprints
Source: eLife. 2019 Apr 24;8:e45133. doi: 10.7554/eLife.45133 (PMC6510536; doi:10.7554/eLife.45133)
Supplement: Figure 1—source data 5. [file elife-45133-fig1-data5.docx]

| **Author name** | **bioRxiv preprints** | **Primary field** | **Email addresses** |
| --- | --- | --- | --- |
| George Davey Smith | 97 | Epidemiology | 4 |
| Ian J. Deary | 61 | Genetics | 4 |
| Andrew M. McIntosh | 57 | Genetics | 1 |
| Mark J. Daly | 47 | Genetics | 3 |
| Richard M. Murray | 45 | Synthetic biology | 2 |
| George M. Church | 43 | Synthetic biology | 4 |
| Wei Wang | 39 | Bioinformatics | 23 |
| Benjamin M. Neale | 39 | Genetics | 2 |
| Alkes L. Price | 36 | Genetics | 1 |
| Jian Yang | 36 | Genetics | 6 |
| Po-Ru Loh | 35 | Genetics | 2 |
| Caroline Hayward | 35 | Genetics | 1 |
| Aarno Palotie | 34 | Genetics | 4 |
| Jay Shendure | 34 | Genomics | 3 |
| Ole A. Andreassen | 34 | Genetics | 2 |

**Figure 1—source data 5:** The top 15 authors with the most preprints on bioRxiv. Names are listed as they appear on biorxiv.org, after making corrections outlined in the Methods section. An author’s "Primary field" is the bioRxiv collection to which they have submitted the most preprints. Preprint count does not account for duplicates: For example, Ian J. Deary and Andrew M. McIntosh are both high on the list, but their counts include multiple preprints that they co-authored together. The "Email addresses" field lists the number of email addresses observed in that author’s preprints that is attributed to them, and is used to approximate the risk that the author is actually a conglomeration of multiple researchers with the same name.
