# Supplementary material for: Tracking the popularity and outcomes of all bioRxiv preprints
Source: eLife. 2019 Apr 24;8:e45133. doi: 10.7554/eLife.45133 (PMC6510536; doi:10.7554/eLife.45133)
Supplement: Figure 1—source data 7. [file elife-45133-fig1-data7.docx]

| **Institution** | **Authors** | **Preprints** |
| --- | --- | --- |
| Stanford University | 1,473 | 1,045 |
| University of Oxford | 1,192 | 902 |
| University of Cambridge | 1,109 | 842 |
| University of Washington | 924 | 609 |
| University College London | 801 | 644 |
| University of Pennsylvania | 764 | 544 |
| University of Michigan | 763 | 484 |
| University of California, San Francisco | 750 | 511 |
| University of California, San Diego | 725 | 495 |
| Imperial College London | 703 | 472 |
| University of Edinburgh | 646 | 487 |
| University of California, Berkeley | 620 | 528 |
| Yale University | 555 | 392 |
| Duke University | 554 | 323 |
| Harvard University | 532 | 557 |
| Harvard Medical School | 529 | 453 |
| Columbia University | 520 | 422 |
| Cornell University | 486 | 365 |
| University of Toronto | 462 | 334 |
| Johns Hopkins University | 461 | 407 |
| University of California, Davis | 461 | 291 |
| Icahn School of Medicine at Mount Sinai | 448 | 281 |
| University of Chicago | 444 | 353 |
| University of British Columbia | 431 | 281 |
| University of Minnesota | 430 | 310 |

**Figure 1—source data 7.** Top 25 institutions with the most authors listing them as their affiliation, and how many papers have been published by those authors. Each institution’s count of total preprints is based on the number of papers posted by authors currently listed with those affiliations, but preprints attributed to authors from multiple institutions count toward the total for all institutions mentioned. A paper with multiple authors from the same institution is counted only once for that institution.
