# Supplementary material for: Tracking the popularity and outcomes of all bioRxiv preprints
Source: eLife. 2019 Apr 24;8:e45133. doi: 10.7554/eLife.45133 (PMC6510536; doi:10.7554/eLife.45133)
Supplement: Figure 4—source data 2. [file elife-45133-fig4-data2.docx]

|  | **2018** | | | **2014 through 2018** | | |
| --- | --- | --- | --- | --- | --- | --- |
| **Journal** | **Total** | **Preprints** | **Proportion** | **Total** | **Preprints** | **Proportion** |
| GigaScience | 89 | 44 | 49.44% | 375 | 90 | 24.00% |
| Genome Biology | 183 | 73 | 39.89% | 1,145 | 181 | 15.81% |
| Genome Research | 169 | 62 | 36.69% | 1,020 | 174 | 17.06% |
| eLife | 1172 | 394 | 33.62% | 6,118 | 750 | 12.26% |
| Nature Methods | 137 | 46 | 33.58% | 847 | 119 | 14.05% |
| PLOS Computational Bio. | 490 | 153 | 31.22% | 3,310 | 337 | 10.18% |
| Nature Genetics | 188 | 55 | 29.26% | 1,169 | 125 | 10.69% |
| G3 | 344 | 95 | 27.62% | 1,962 | 246 | 12.54% |
| Genetics | 276 | 71 | 25.72% | 1,899 | 269 | 14.17% |
| Bioinformatics | 829 | 209 | 25.21% | 4,694 | 472 | 10.06% |
| PLOS Genetics | 515 | 117 | 22.72% | 3,885 | 292 | 7.52% |
| Molecular Bio. and Evolution | 228 | 47 | 20.61% | 1,544 | 157 | 10.17% |
| PLOS Biology | 333 | 67 | 20.12% | 1,379 | 108 | 7.83% |
| Genome Bio. and Evolution | 199 | 40 | 20.10% | 1,571 | 109 | 6.94% |
| Molecular Bio. of the Cell* | 215 | 38 | 17.67% | 2,012 | 88 | 4.37% |
| mBio | 373 | 64 | 17.16% | 2,383 | 108 | 4.53% |
| NeuroImage | 862 | 117 | 13.57% | 5,186 | 214 | 4.13% |
| Biophysical Journal* | 482 | 65 | 13.49% | 3,103 | 109 | 3.51% |
| BMC Bioinformatics | 456 | 61 | 13.38% | 3,176 | 137 | 4.31% |
| Journal of Neuroscience | 777 | 98 | 12.61% | 6,569 | 172 | 2.62% |
| Development | 360 | 38 | 10.56% | 2,296 | 90 | 3.92% |
| Molecular Ecology | 315 | 32 | 10.16% | 2,245 | 92 | 4.10% |
| Nucleic Acids Research | 1142 | 115 | 10.07% | 7,896 | 265 | 3.36% |
| BMC Genomics | 904 | 75 | 8.30% | 6,366 | 174 | 2.73% |
| Nature Communications | 4979 | 355 | 7.13% | 22,338 | 529 | 2.37% |
| PNAS | 3195 | 212 | 6.64% | 19,697 | 432 | 2.19% |
| Cell Reports | 1230 | 79 | 6.42% | 6,846 | 136 | 1.99% |
| PeerJ | 1877 | 53 | 2.82% | 7,047 | 120 | 1.70% |
| Scientific Reports | 16899 | 398 | 2.36% | 9,7451 | 827 | 0.85% |
| PLOS ONE | 17021 | 395 | 2.32% | 138,009 | 741 | 0.54% |

**Figure 4—figure supplement 1.** The total number of articles published by the top 30 journals that have published the most bioRxiv preprints, compared to how many of those articles appeared on bioRxiv. The list is ordered by the proportion of 2018 publications first appeared on bioRxiv. Only 2018 and the total number are displayed here; annual data from 2014 through November 2018 is available in the source data. Total article counts are from the number of works in the “article” category as indexed by Web of Science (Clarivate Analytics). Journals marked with an asterisk have a large number of published works categorized on Web of Science as “meeting abstracts”; for consistency, those are not included in the counts here, though it is possible some of the preprints published by these journals fall into that category.
